# Gamma’ fibrinogen levels as a biomarker of COVID-19 respiratory disease severity

**DOI:** 10.21203/rs.3.rs-2160004/v1

**Published:** 2022-10-21

**Authors:** Lucy Z. Kornblith, Bindhya Sadhanandhan, Sreepriya Arun, Rebecca Long, Alicia J. Johnson, Jamie Noll, C. N. Ramchand, John K. Olynyk, David H. Farrell

**Affiliations:** University of California; Theragen Biologics Pvt Ltd; Theragen Biologics Pvt Ltd; Fiona Stanley Fremantle Hospital Group, Murdoch, Western Australia, Australia and Edith Cowan University, Joondalup, Western Australia, Australia; Oregon Health & Science University; Gamma Diagnostics; MagGenome Pvt Ltd; Fiona Stanley Fremantle Hospital Group, Murdoch, Western Australia, Australia and Edith Cowan University, Joondalup, Western Australia, Australia; Oregon Health & Science University

**Keywords:** Biomarkers, COVID-19, Severity

## Abstract

Coronavirus disease 2019 (COVID-19) is characterized by a pro-inflammatory state associated with organ failure, thrombosis, and death. We investigated a novel inflammatory biomarker, γ’ fibrinogen (GPF), in 103 hospitalized patients with COVID-19 and 19 healthy controls. We found significant associations between GPF levels and the severity of COVID-19 as judged by blood oxygen saturation (SpO_2_). The mean level of GPF in the patients with COVID-19 was significantly higher than in controls (69.8 (95% CI 64.8–74.8) mg/dL compared with 36.9 (95% CI 31.4–42.4) mg/dL, p < 0.0001), whereas C-reactive protein (CRP), lactate dehydrogenase (LDH), and total fibrinogen levels were not significantly different between groups. Mean GPF levels were significantly highest in patients with severe COVID-19 (SpO_2_ ≤ 93%, GPF 75.2 (95% CI 68.7–81.8) mg/dL), compared to mild/moderate COVID-19 (SpO_2_ >93%, GPF 62.5 (95% CI 55.0–70.0) mg/dL, p = 0.01, AUC of 0.68, 95% CI 0.57–0.78; Youden’s index cutpoint 62.9 mg/dL, sensitivity 0.64, specificity 0.63). In contrast, CRP interleukin-6, ferritin, LDH, D-dimers, and total fibrinogen had weaker associations with COVID-19 disease severity (all ROC curves with lower AUCs). Thus, GPF may be a useful inflammatory marker of COVID-19 respiratory disease severity.

## Introduction

Coronavirus disease 2019 (COVID-19) is a viral respiratory disease that results in high levels of pro-inflammatory and pro-thrombotic biomarkers that are associated with worse outcomes, including death^1^. Elevated levels of C-reactive protein (CRP), interleukin-6 (IL-6), ferritin, lactate dehydrogenase (LDH), D-dimer, and fibrinogen have suggested various potential mechanisms in the pathogenesis of poor outcomes in COVID-19^2^. For example, elevated levels of CRP ferritin, and fibrinogen indicate activation of the *acute phase response*, while elevated circulating D-dimer levels suggest that fibrinolytic pathways are likely intact and dissolving fibrin^3^. Despite this, fibrin deposits in lungs and other organs suggests dysregulation of balance in fibrin-forming (i.e., thrombin generation) and dissolving (i.e., plasmin generation) pathways, and autopsies of patients with COVID-19 show the presence of thrombosis in up to 60% of the patients^4^.

γ’ fibrinogen (GPF) is a novel inflammatory biomarker that has been associated with cardiovascular^5^ and inflammatory^6^ diseases. We have previously shown that extraordinarily high levels of GPF in a small population of patients with COVID-19 were associated with the need for extracorporeal membrane oxygenation and death^7^. We therefore tested the hypothesis that GPF levels can stratify COVID-19 respiratory disease severity in a larger population of patients.

## Patients And Methods

### Study Participants

This study was approved by the Institutional Ethical Committee at the Micro Therapeutics Laboratory, Chennai, India and Saveetha Medical College, Chennai, India. All participants provided written informed consent.

#### Patients with COVID-19

Patients were recruited from Saveetha Hospital. Inclusion criteria were hospitalized patients aged 18–65 years and laboratory confirmation of infection with SARS-CoV-2 by positive reverse transcription polymerase chain reaction (RT-PCR). Exclusion criteria were pregnant or lactating women, participation in any interventional drug clinical study during the study, or a known inflammatory condition. Patients were enrolled between June 14, 2021 and February 21, 2022. Blood samples were obtained and clinical characteristics were determined at admission.

#### Healthy controls

Inclusion criteria were healthy volunteers age 18–65 years with laboratory confirmation of non-infection with SARS-CoV-2 by negative RT-PCR. Exclusion criteria were known bleeding disorders, liver or kidney disease, cancer, history of surgery or thrombotic event within the past 3 months, previous history of viral infection and other diseases, or participation in any other study.

### Laboratory Testing

Subjects had 2 ml of whole blood collected in 3.2% sodium citrate at admission, which was processed into plasma and banked at −80°C. Ferritin, D-dimer, and IL-6 were measured using chemiluminescence (Maglumi), CRP using tubidimetrics (Turbilatex), LDH using a latex reagent (Mindray), and total fibrinogen using a clot-based auto chemistry analyzer (Beacon Diagnostics PVT LTD). Cycle threshold (Ct) values for (RT-PCR) were obtained from the Micro Therapeutics Laboratory and Saveetha Medical College. GPF was analyzed using a commercial enzyme-linked immunoassay (Gamma Coeur ELISA Kit - GCEK001, Zeus Scientific).

### Statistical Analysis

Severity of COVID-19 was defined as mild/moderate (SpO_2_ >93%) or severe (SpO_2_ ≤ 93%) on room air measured at admission^8^. Differences in GPF levels among patients with mild/moderate or severe COVID-19 were analyzed using ANOVA with multiple comparisons (insert statistical package here - I used Prism 9, GraphPad Software, LLC). We examined the relationship between COVID-19 severity and other biomarkers using a Wilcoxon rank sum test for skewed biomarkers (IL-6, LDH, D-dimer, ferritin, and CRP), and a t-test for normally-distributed biomarkers (total fibrinogen). These methods were applied to examine the differences between patients with COVID-19 and controls as well. Prior to running t-tests, an F-test for the equality of variances was run. If unequal variances were found, unequal variance t-tests were run. Otherwise, equal variance t-tests were used.

ROC curves were used to determine the ability of GPF to differentiate between patients with COVID-19 and controls, as well as between patients with severe COVID-19 and mild/moderate COVID-19. Area under the curve (AUC) and 95% confidence intervals (CI) were calculated. For each of these curves, Youden’s index was calculated to determine the GPF cutpoint that maximized sensitivity and specificity. AUC and CI were also calculated for the other biomarkers. Within patients with COVID-19, correlation between GPF and other biomarkers was calculated using Spearman’s rho due to the skewed distribution of most of the biomarkers. All statistical analyses were performed using Stata 16 (StataCorp, College Stations, TX).

## Results

The general characteristics of the COVID-19 and health control participants are shown in Table 1.

### Biomarker Comparison between Patients with COVID-19 and Healthy Controls

**Figure 1:**
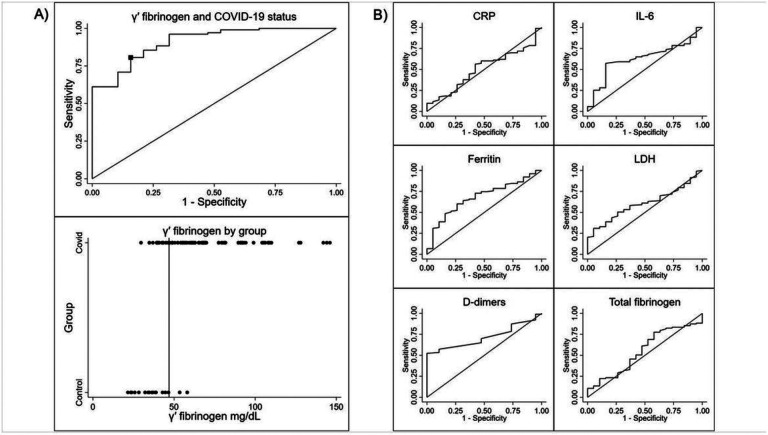
ROC curve and Youden’s index to determine association of COVID-19 with GPF level, and six other biomarkers. A) The AUC for GPF was 0.91 (95% CI: 0.85–0.98). Youden’s index cutpoint was 46.9 mg/dL between healthy controls and patients with COVID-19. The sensitivity at this cutpoint was 0.81 and the specificity was 0.84. B) The AUC for CRP was 0.51 (95% CI: 0.37–0.64). The AUC for IL-6 was 0.63 (95% CI: 0.51–0.75). The AUC for ferritin was 0.68 (95% CI: 0.56–0.80). The AUC for LDH was 0.61 (95% CI: 0.49–0.72). The AUC for D-dimer was 0.72 (95% CI: 0.62–0.81). The AUC for total fibrinogen was 0.55 (95% CI: 0.41–0.70).

The mean GPF in patients with COVID-19 was 69.8 mg/dL, compared to 36.9 mg/dL in controls (mean difference: 32.9 mg/dL [95% CI: 25.6–40.2]; p < 0.0001). We also observed significant differences in median ferritin (p = 0.01) and median D-dimer levels (p = 0.003). Table 2 summarizes the biomarker comparisons by COVID-19 status. Using ROC curves, GPF differentiated between COVID-19 patients and controls very well (AUC 0.91, 95% CI: 0.85–0.98; Youden’s index cutpoint 46.9 mg/dL, sensitivity 0.81, specificity 0.84; Fig. 1A), and all other biomarkers had lower AUC (Fig. 1B).

### Biomarker Comparisons with GPF Levels

Spearman’s rho analysis showed that GPF levels in patients with COVID-19 were not significantly correlated with IL-6 (rho = 0.11, p = 0.28), ferritin (rho = 0.04, p = 0.72), D-dimer (rho = 0.18, p = 0.069), or fibrinogen (rho = 0.19, p = 0.051), but were significantly correlated with CRP (rho = 0.23, p = 0.019; rho = 23) and LDH (rho = 0.27, p = 0.0064; rho = 0.27), although the rho values do not suggest a robust association.

### Biomarker Comparison with COVID-19 Disease Severity

**Figure 2:**
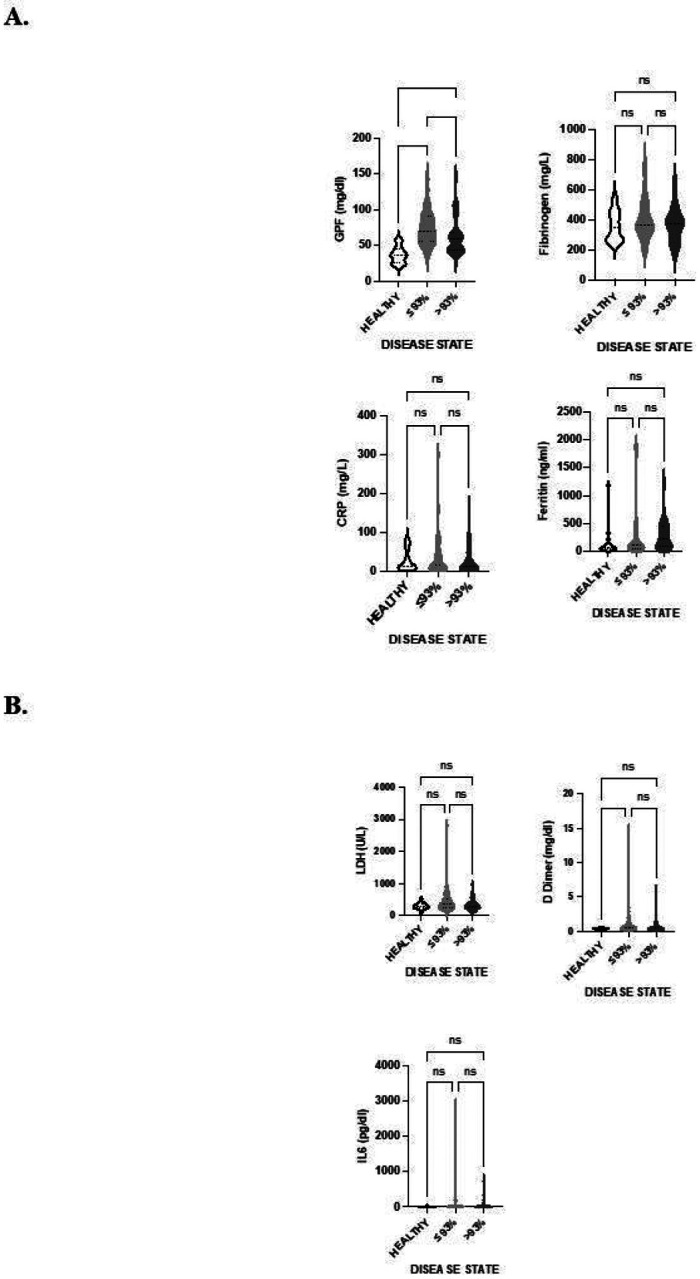
Pairwise comparisons between biomarkers in healthy controls, mild/moderate COVID-19 patients, and severe COVID-19 patients. We examined the relationship between COVID-19 severity and other biomarkers using a Wilcoxon rank sum test for skewed biomarkers (GPF, IL-6, LDH, D-dimer, ferritin, and CRP), and a t-test for normally-distributed biomarkers (total fibrinogen).

GPF was significantly associated with severe (mean = 75.2 mg/dL) vs. mild/moderate (mean = 62.5 mg/dL) COVID-19 severity (mean difference: 12.8 mg/dL [95% CI: 2.8–22.7]; p = 0.012), as defined by SpO_2_ levels ≥ 93% (mild/moderate) or < 93% (severe). Pairwise comparisons between the healthy controls and either mild/moderate or severe COVID-19 patients showed significant differences (Fig. 2a). In addition, there was a significant difference in GPF levels between mild/moderate *vs*. severe COVID-19 patients. In contrast, none of the other biomarkers measured, neither total fibrinogen, CRP ferritin (Fig. 2a), LDH, D-dimer, nor IL-6 (Fig. 2b) showed significant differences between all three groups. D-dimers did show a significant difference between the healthy controls and the severe COVID-19 patients, but not between the healthy controls and mild/moderate COVID-19 patients nor between mild/moderate *vs*. severe COVID-19 patients.

**Figure 3:**
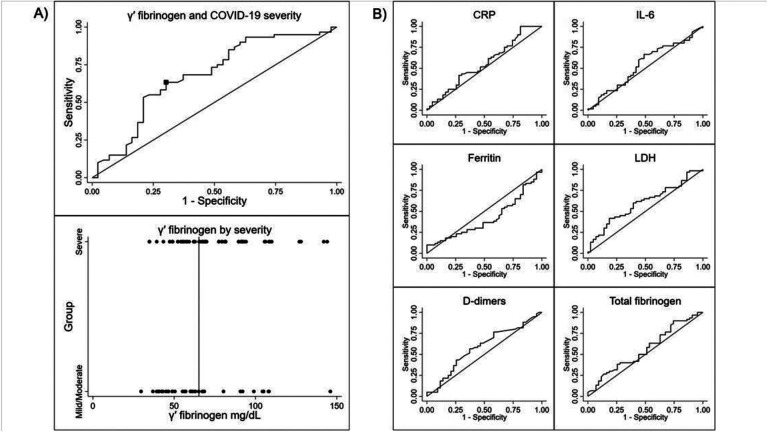
Relationship between COVID-19 severity, GPF level, and six other biomarkers. COVID-19 severity was determined by SpO_2_ levels, with mild/moderate defined as SpO_2_ >93%, and severe as SpO_2_ ≤ 93%. A) The AUC for GPF was 0.68 (95% CI: 0.57–0.78). Youden’s index cutpoint was 62.9 mg/dL between mild/moderate *vs*. severe COVID-19. The sensitivity at this cutpoint was 0.64 and the specificity was 0.63. B) The AUC for CRP was 0.56 (95% CI: 0.44–0.67). The AUC for IL-6 was 0.54 (95% CI: 0.43–0.66). The AUC for ferritin was 0.42 (95% CI: 0.31 −0.53). The AUC for LDH was 0.60 (95% CI: 0.49–0.71). The AUC for D-dimer was 0.58 (95% CI: 0.47–0.70). The AUC for total fibrinogen was 0.56 (95% CI: 0.45–0.68).

Using ROC curves, GPF differentiated between mild/moderate *vs*. severe COVID-19 patients (AUC 0.68, 95% CI: 0.57–0.78; Youden’s index cutpoint of 62.9 mg/dL, sensitivity 0.63, specificity 0.63; Fig. 3A). None of the other biomarkers were significantly associated with severity (p > 0.05; Fig. 3B), and they all had lower AUC.

## Discussion

We found that GPF levels were increased significantly over controls in hospitalized patients with COVID-19, and had stronger associations with severity of COVID-19 respiratory disease compared to several other inflammatory biomarkers, including CRP IL-6, ferritin, LDH, D-dimer, and total fibrinogen. Hence these findings have potential clinical implications suggesting that elevated GPF levels may be useful as a biomarker for respiratory disease severity in COVID-19. Furthermore, GPF levels did not significantly correlate with IL-6, ferritin, D-dimer, or total fibrinogen. These findings suggest that the associations between COVID-19 and COVID-19 severity with GPF levels cannot simply be due to total fibrinogen levels.

**Figure 4:**
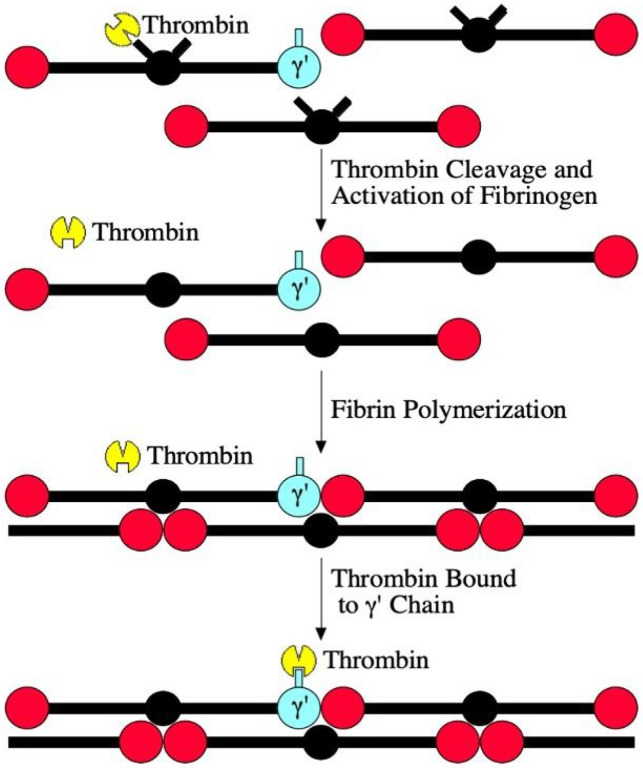
Model of GPF mechanism of thrombosis. The model starts with free thrombin cleaving fibrinopeptides A and B from all forms of fibrinogen *via* its active site (cutout triangle). This converts the fibrinogen to fibrin, which polymerizes into an insoluble clot. The free thrombin can then bind to GPF *via* thrombin anion-binding exosite II (cutout square), concentrating active thrombin on the growing fibrin clot. This bound thrombin is resistant to heparin, since heparin binds to anion-binding exosite II, which is blocked by the g’ chain.

These findings have potential clinical implications regarding prophylactic anticoagulation of COVID-19 patients. The resistance of GPF-bound thrombin (Fig. 4) to heparin suggests that heparin prophylaxis may be less effective than treatment with other anticoagulants, particularly direct thrombin inhibitors. In point of fact, in critically ill patients with COVID-19, therapeutic-dose heparin did not result in a greater probability of survival to hospital discharge or a greater number of days free of cardiovascular or respiratory organ support^9^. It is possible that inhibition of factor Xa by current DOACs may also reduce the levels of active thrombin and thereby prevent activation of thrombin substrates by GPF-bound thrombin, including factor V, factor VIII, factor XI, factor XIII, and fibrinogen, as well as platelet substrates such as PAR-1 and PAR-4. In addition, it is also possible that warfarin anticoagulation may be effective at preventing thrombosis due to GPF-bound thrombin by reducing the levels of active vitamin K-dependent coagulation factors, and therefore, generation of thrombin.

Our study has limitations, in that this was a small observational study without outcome data. It is known that comorbidities including heart disease, hypertension, and diabetes in patients with COVID-19 increase the risk for severe disease and mortality^10–13^. These could confound the identified association of COVID-19 severity with levels of GPF. GPF (and indeed the other markers) was measured by a single assay, and thus the findings may not hold true for other assay methods. GPF assays are not available in most labs, unlike the other assays, but a commercial ELISA is now available.

In conclusion, our study suggests that GPF may be a valuable biomarker for assessing COVID-19 respiratory disease severity. Evidence is lacking as to the causal mechanisms or whether the associations are specific to effects of COVID-19 infection or the consequences of a systemic inflammatory response. However, recent *in vitro* evidence demonstrates that high GPF levels in patient plasma increase clot formation at both arterial and venous shear conditions *in vitro*^14^. Future investigations of GPF as a driver of thromboinflammation and poor outcomes in COVID-19, and as a biomarker for other inflammatory diseases, both infectious and non-infectious, should be pursued.

## Figures and Tables

**Table 1:** Baseline demographic and biochemical characteristics of study patients.

Variable	COVID-19 (N = 103)	Controls (N = 19)
Age - (years) Mean (SD)	49.2 (14.05)	37.3 (11.74)
Male- n (%)	59 (57.3)	10 (52.6)
Female- n (%)	44 (42.7)	9 (47.4)
BMI - (kg/m^2^) Mean (SD)	22.2 (3.87)	24.3 (2.85)
Body temperature (°C) - Mean (SD)	37.6 (0.51)	36.6 (0.26)
SpO_2_ - (%) Median (Q1, Q3)	93 (92, 94)	98 (96, 99)
Pulse rate - (bpm) Median (Q1, Q3)	79 (75, 83)	72 (70, 73)
Respiratory rate - (breaths per minute) Mean (SD)	22.7 (1.78)	20.7 (0.75)
Systolic BP - (mm Hg) Mean (SD)	123.7 (10.87)	119.7 (4.81)
Diastolic BP - (mm Hg) Mean (SD)	80.5 (9.52)	82.3 (2.69)

**Table 2:** Biomarkers

Variable	COVID-19 (N = 103)	Controls (N = 19)	p-value
GPF (mg/dL) - Mean (SD)	69.8 (25.66)	36.9 (11.47)	<0.0001
CRP (mg/L) - Median (Q1, Q3)	14.7 (4.1, 47.3)	10.1 (5.8, 49.7)	0.9241
IL-6 (pg/mL) - Median (Q1, Q3)	10.8 (6.1, 60)	6.9 (5.2, 8.1)	0.0712
Ferritin (ng/mL) - Median (Q1, Q3)	139 (66, 402.7)	71 (49, 133)	0.0128
LDH (U/L) - Median (Q1, Q3)	350.9 (244, 486.5)	291 (244, 358)	0.1458
D-dimer (mg/L) - Median (Q1, Q3)	0.5 (0.3, 0.96)	0.3 (0.2, 0.4)	0.0026
Total fibrinogen (mg/dL) - Mean (SD)	386.0 (128.5)	357.7 (99.40)	0.3661

## Data Availability

The datasets generated during and/or analyzed during the current study are available from the corresponding author on reasonable request.
